# The Royal Hospital for Incurables

**Published:** 1894-01-13

**Authors:** 


### THE ROYAL HOSPITAL FOR INCURABLES.

### VOICES FROM THE INTERIOR.

WE have much pleasure in publishing the following communication, which purports to be written on behalf of the inmates of this institution. It carries with it internal evidence, which warrants us in applying to it the words of Isaac of old, "The voice is Jacob's voice, but the hands are the hands of Esau."—ED. THE HOSPITAL.

May we request you in the cause of justice and humanity to publish this letter in your valuable paper, THE HOSPITAL. We read your articles in the same with surprise and regret —surprise that you should seek the destruction of our home, and regret that you should misunderstand us and our requirements.

You infer that we should be better off either with our own friends or in the workhouse. We beg to differ. Nine-tenths of us have no homes to go to, and ninety out of each hundred would rather starve to death in a third floor back attic than herd with the scum of the earth in a workhouse.

We have neither sought or received special favours here; therefore, from an unprejudiced standpoint, we are able to form a calm and uninstructed opinion. We can truthfully assert that the Royal Hospital for Incurables was never in such good order or cleanliness as during the last few years, and numerous improvements have been experienced.

Unfortunately there are some chronic grumblers amongst us whom not even the angel Gabriel could satisfy, and these would be the first to complain if changes did not please them. Others have weakened intellects and are incapable of forming an opinion. The doctor who visits us daily is a man of experience. Should we have a resident doctor, doubtless he would be a young man fresh from the hospitals, who would torture us chronics with experiments, spend his time smoking cigars and turning the house upside down until he discovered that he had misunderstood the patient's real requirements, then, in despair, make his exit at the end of six months, to be succeeded by some old gentleman past out-door practice, who would take about an hour to arrive at an opinion. We should look on with dismay at such an unpromising outlook.

No change is necessary amongst the nurses; they are kind and attentive, the head ones being highly trained and up to all the latest requirements.

The gentlemen of the committee come amongst us to question and hear any complaints, and if people will but have patience with them they will move on with the times in all things that are necessary and desirable.

The letter which you quote concerning the branch home at St. Leonards is practically correct. Mrs. Bartholomew is all that is therein stated, but we would add that, having only ten patients in her care, it is part of her duty to be a companion to them, whereas the Matron of this hospital has 220 patients to deal with, also a large staff of paid officials. It would be impossible for her to devote her time to us individually, neither is it necessary; we are quite capable of amusing ourselves with the aid of our numerous lady and gentlemen visitors.

We are neither persecuted or unhappy, and we invite the public to come and look at us, then they can decide for themselves, also pass an opinion upon our dinners, which we are sure would frequently be good enough for even the members of the House of Lords' Committee.

